# Mass balance, metabolic disposition, and pharmacokinetics of a single IV dose of [14C]CA102N in HT-29 xenograft athymic nude mice

**DOI:** 10.3389/fphar.2024.1440679

**Published:** 2024-12-05

**Authors:** Eskouhie Tchaparian, Hua-Yang Lin, Yuchih Chen, J. Neil Hunter, Sindy Yin, Huey Ng, Albert Wu

**Affiliations:** ^1^ Holy Stone Healthcare, Preclinical and Development Div Hsinchu, Taipei, Taiwan; ^2^ MDT Int’l SA, Geneva, Switzerland

**Keywords:** hyaluronic acid, distribution, excretion, metabolism, pharmacokinetics, cancer therapy

## Abstract

**Introduction:**

CA102N is a novel anticancer drug developed by covalently linking H-Nim (N-(4-Amino-2-phenoxyphenyl methanesulfonamide) to Hyaluronic Acid to target CD44 receptor-rich tumors. The proposed approach seeks to enhance the efficacy and overcome limitations associated with H-Nim, including poor solubility and short half-life.

**Methods:**

The study aimed to evaluate the pharmacokinetics, biodistribution, metabolism, and tumor permeability of [14C] CA102N in xenograft mice following a single intravenous dose of 200 mg/kg. Liquid scintillation counting analysis was used for the pharmacokinetics and mass balance analysis. Metabolite profiling was assessed by HPLC-MS coupled with a radio flow-through detector. Quantitative Whole-Body Autoradiography was used to determine tissue distribution. Concentrations of CA102N and its metabolites were measured using total radioactivity data from urine, feces, and tissue samples.

**Results:**

About 94.9% of the administered dose was recovered at 240 h post-dose. The primary route of radioactivity elimination was through urine, accounting for an average of 77% of the dose with around 13.2% excreted in the feces. Tissue distribution showed rapid accumulation within 0.5 h post-administration, followed by a fast decline in most tissues except for the tumor, where slow elimination was observed. CA102N/metabolites exhibited a two-phase pharmacokinetic profile, characterized by an initial rapid distribution phase and a slower terminal elimination, with a half-life (t_1/2_) of 22 h. The mean maximum concentration (C_max_) of 1798.586 µg equivalents per ml was reached at 0.5 h (T_max_). Most of the radioactivity in plasma was attributed to CA102N, while small-molecule hydrolysis products dominated the excreta and tissue samples. Metabolite profiling revealed two major hydrolysis products: H-Nim-disaccharide and H-Nim-tetrasaccharide. No unchanged [14C] CA102N was detected in urine or feces, suggesting that CA102N undergoes extensive metabolism before excretion.

**Conclusion:**

The current data provided valuable insights into the pharmacokinetics, metabolism, and tissue/tumor distribution of CA102N in mice. These findings demonstrated that metabolic clearance is the primary elimination pathway for CA102N and that the drug exhibits tumor retention, supporting its development as an anticancer therapy. Our results provided a strong pharmacological basis for the advancement of CA102N into the clinic.

## 1 Introduction

In the past decade, there has been growing interest in the development of drug delivery systems to improve the pharmacological and therapeutic properties of parenterally administered drugs. Hyaluronic acid (HA)-based delivery systems have gained significant attention recently, especially for their potential in cancer therapy (Jia et al., 2022; Luo et al., 2019).

HA is a naturally occurring glycosaminoglycan found in the extracellular matrix of connective tissues. Its unique properties make it a promising and versatile drug delivery system. HA is a biocompatible, biodegradable, non-toxic, non-immunogenic polymer, with a high affinity to the cluster of differentiation 44 (CD44) receptor which is overexpressed in various tumor cells and plays a key role in tumor progression (Blundell et al., 2004; Kalaydina et al., 2018; Pérez-Herrero and Fernández-Medarde, 2015; Elzahhar et al., 2019; Li et al., 2017; Soppimath et al., 2001; Almawash et al., 2018; Su et al., 2011; Xu et al., 2015).

The newly developed Hyaluronic Acid Drug Conjugate (HADC) platform employs the natural properties of HA to deliver drugs to target sites, such as tumors. (Mattheolabakis et al., 2015; Saravanakumar et al., 2014). By exploiting HA’s binding affinity to CD44, HADC can deliver therapeutic agents directly to diseased or cancer tissues, thus improving efficacy while reducing off-target effects and toxicity. Additionally, with its biocompatibility, biodegradability, and ability to enhance drug solubility, HADC could be an ideal platform for creating efficient and targeted drug delivery system.

CA102N is a novel HADC molecule, consisting of HA in its sodium salt form coupled to H-Nim (N-(4-Amino-2-phenoxyphenyl, methanesulfonamide), a derivative of cyclooxygenase-2 (COX2) inhibitor Nimesulide (Nim) (Jian et al., 2017). CA102N is currently under development in clinical phase II studies for metastatic colorectal cancer. Cyclooxygenase COX-2 is an inducible enzyme that catalyzes the metabolism of Prostaglandin E2 (PGE2). Studies have shown that COX-2–derived PGE2 promotes angiogenesis and tissue invasion of tumors, resistance to apoptosis, and suppression of immune responses in cancer (FitzGerald, 2011; Liu et al., 2015; Sheng et al., 2020); moreover, inhibition of COX-2 has been implicated in the treatment and prevention of various human cancers (Evans and Kargman, 2004). The anticancer activities of Nim have been shown in several studies (Okajima et al., 1998; Fukutake et al., 1998; Zhong et al., 2012; Li et al., 2003; Liang et al., 2009); however, its poor solubility, short t_1/2_, and association with increased risk for hepatotoxicity restrict its application of becoming a lead anticancer drug (Wei et al., 2021; Neha et al., 2012). CA102N was developed to address these limitations by specifically delivering H-Nim to cancer cells (Mero and Campisi, 2014). Pharmacological studies have demonstrated the apoptotic and tumor growth inhibitory effects of CA102N. Administration of 200 or 400 mg/kg CA102N) weekly (QW) or three times a week (TIW) in HT-29 xenograft nude mice led to a significant reduction in tumor burden compared to vehicle-treated controls (Lin et al., 2017).

Pharmacokinetics, distribution, metabolism, and excretion (PK-DME) characteristics of a novel molecule are critical for efficacy, safety, and ultimately success in the clinic. Further, the assessment of PK-DME data and tumor-targeting properties of cancer therapeutics allows for a more robust nonclinical evaluation. Therefore, the primary objective of this study was to assess the PK-DME properties of CA102N, using a human colon carcinoma (HT-29) xenograft model. This model was chosen for its ability to provide more relevant and translational cancer research data. Quantitative Whole-Body Autoradiography (QWBA) and [14C] labeling were employed to comprehensively evaluate the pharmacokinetics and tumor-targeting attributes of CA102N. Further, the use of the radiolabeled ([14C]CA102N) enabled *in vivo* tracking of the drug and its metabolites and highlighted the clearance mechanism.

The findings of this investigation were used to recommend clinical studies as well as to reduce uncertainties in dosing and safety, which are key aspects of translational research.

## 2 Materials and methods

### 2.1 Test article and General chemicals

#### 2.1.1 Test articles

CA102N (N-(4-Amino-2-phenoxyphenyl) methanesulfonamide-graft-poly (β-D-(1, 3) glucuronic acid-β-D-(1, 4)-N-acetylglucosamine) conjugate (purity 100%) and H-Nim were synthesized in the Drug Discovery Laboratory of Holy Stone Healthcare (Hsinchu, Taiwan). CA102N is a white or off-white fibrous crystalline aggregate, sparingly soluble in water, and insoluble in ethanol.

[14C]-CA102N ([aniline ring-14C(U)]) (purity 97.7%), was supplied by Moravek Biochemicals, Inc. (Brea CA). The synthesis of radiolabelled CA102N was designed to provide a [14C]-CA102N [aniline ring-14C(U)]- labeling on H-Nim. The test article was a mixture of [14C]CA102N [aniline ring-14C(U)] and CA102N, (purity 100%). The structure of the test article is presented in Supplementary Figure S1, the asterisk indicates the position of the 14C label.

[14C]-CA102N was supplied as a solution in water with a specific activity of 5.95 μCi/mg. The solution was stored at 5°C, protected from light. The radiopurity and stability of [14C]CA102N in the dosing formulation were assessed by radio-HPLC, (Supplementary Figure S2).

#### 2.1.2 General chemicals

Formic acid, HPLC-grade acetonitrile, and HPLC-grade methanol, Dulbecco’s Phosphate-Buffered Saline (DPBS) were purchased from Fisher Scientific (Pittsburgh, PA). Type I water was generated using an Elgastat UHQ PS water purification system. The reference standards H-Nim saccharide and H-Nim disaccharide were synthesized by ACME (Palo Alto, CA). The remaining reagents used in the study were commercially sourced and of analytical-grade quality.

### 2.2 Dosing formulation

The CA102N dosing formulation was prepared on the day of dosing by mixing an appropriate quantity of [14C]CA102N and CA102N in the vehicle, DPBS. The target CA102N formulation concentration was 40 mg/mL and the radioactivity concentration was approximately 40 μCi/mL resulting in the target dose of 200 mg/kg and approximately 200 μCi/kg, or a dose volume of 5 ml/kg/dose. CA102N was mixed thoroughly until the test article was completely dissolved and continuously stirred during dosing and sampling procedures. Prior to dose administration, formulations were filtered using 0.22 μm filters to ensure sterility.

### 2.3 Test animals and tissue collection

The animal studies were conducted in compliance with the protocols approved by the Institutional Animal Care and Use Committee (IACUC). For studies conducted at QPS (Newark, DE), the IACUC Protocol Number was 004, and the Study Numbers were 975N–1602 and 975N-1601. For studies performed at the National Chung Hsing University (Taichung, Taiwan) the IACUC Protocol Number was 110-036.

Female BALB/c athymic (nu+/nu+) mice (n = 48) were generated by Taconic (Germantown, NY). Mice (18–22 g) were acclimated for at least 3 days before subcutaneous implantation of 1 × 10^7^ HT-29 colorectal carcinoma (ATCC number HTB38) cells in 100 μL 50:50 matrigel: PBS. Tumors were allowed to grow to 200–500 mm^3^ sizes before initiating the study. Each animal was assigned a permanent identification number, and identified with a tail mark before being placed on the study. Food and water were provided *ad libitum*. Animal rooms were controlled to maintain a temperature of 68°F–79°F, and 20%–70% relative humidity, with a 12 h light/12 h dark cycle. Observations were recorded for all animals pre-dose and at least once daily post-dose. Mice were randomized into 3 groups and housed (n = 3 per cage) in metabolism cages (Group 1, n = 9) or shoebox cages (Group 2, n = 29 and Group 3, n = 10). Each mouse received a single bolus injection of CA102N via the tail vein. Urine, feces, cage residue, blood/plasma, tissue samples, and carcasses were collected from different groups for Mass Balance (MB), Metabolite Identification (MET-ID), Pharmacokinetics (PK), and Quantitative Whole-Body Autoradiography (QWBA) studies.

Samples were collected on days 0 through 10. For the MET-ID studies, complete urine, feces, and cage residue specimens were collected (up to 120 h) over dry ice from each metabolism cage (n = 3 mice/cage, Group 1). Blood samples were collected via cardiac puncture (Group 2), into EDTA-containing tubes at 0 (pre-dose), 0.5, 2, 8, 24, 48, 72, 120,168, 240, and 336 h after the dose, and plasma samples were harvested on wet ice for PK analysis. Tumor and liver samples were also collected for detailed metabolites study at 240 h post-dose. The weights of all samples (except blood and plasma) were recorded and then stored at −70°C for later analysis. For the QWBA study (Group 3), mice were euthanized by being frozen in a hexane/solid carbon dioxide bath, blood samples were collected, and the carcasses (0 (pre-dose), 0.5, 2, 8, 24, 48, 72, 120,168, 240 and 336 h after dose) were stored at −20°C before processing for QWBA analysis.

### 2.4 Radioactivity measurement, liquid scintillation counting (LSC), analysis of mass balance samples, blood, and plasma

Radioactivity in diluted formulations, plasma, and urine, was quantitated by mixing with a liquid scintillator, Ultima Gold scintillation cocktail (5 mL, PerkinElmer). Samples were analyzed by LSC for total radioactivity content. Solid samples were homogenized (3 X volume of distilled water) and placed in a Combusto-Cone^®^ that contained a Combusto-Pad^®^ to dry overnight before combustion in a sample oxidizer (Perkin Elmer Model 307). The resulting 14CO_2_ liberated during combustion was trapped in CarboSorb^®^ and Perma-Fluor^®^ scintillation cocktail was added automatically to the samples before total radioactivity measurement by LSC.

All LSC analyses were performed using a liquid scintillation analyzer (Perkin Elmer Model 2800TR or Model 2900TR). The LSC data in CPM were automatically corrected for counting efficiency using an external standardization technique and an instrument-stored quench curve generated from sealed quenched standards, to obtain disintegrations per minute (dpm). Data were corrected to the background by subtracting the dpm value measured from the analysis of blank samples (cocktail only).

The extraction recovery of all samples was evaluated by mixing with MeOH: H2O (1:1, v/v). (except urine samples which were evaluated for centrifugation recovery). The mixtures were centrifuged and the supernatants’ radioactivity was determined by LSC. The % extraction recoveries were determined in all the samples.

### 2.5 Sample analysis for QWBA

The pinna, limbs, and tail were removed from each frozen carcass (Group 3) and the remaining carcass was embedded in an aqueous suspension of approximately 1% (w/v) carboxymethylcellulose and frozen into a block. The blocks were stored at approximately −20°C before sectioning. Each blocked carcass was mounted on a microtome stage (Leica CM3600 Cryomacrocut and/or a Vibratome 9800, Leica Corp., Nussloch, Germany) and maintained at approximately −20°C. Sections were collected and allowed to dry by sublimation in the cryomicrotome at −20°C for at least 48 h. A set of sections for each mouse was mounted on a cardboard backing, covered with a thin plastic wrap, and exposed along with calibration standards of [14C]-glucose mixed with blood at 10 different concentrations to a [14C]-sensitive phosphor imaging plate (Fuji Biomedical, Stamford, CT). The imaging plates and sections were placed in light-tight exposure cassettes, for a 4-day (96 h) exposure at room temperature. The imaging plates were scanned using the GE Healthcare Typhoon FLA 9500 image acquisition system (GE/Molecular Dynamics, Sunnyvale, CA, United States). Quantification was performed by image dosimetry using MCID image analysis software (Version 7.0, Interfocus Imaging Ltd., Linton, Cambridge, United Kingdom). A standard curve was constructed from the integrated response (MDC/mm2) and the nominal concentrations of the [14C]-calibration standards. The concentrations of radioactivity were expressed as the µg equivalents of CA102N per gram sample (µg equiv/g).

### 2.6 *In Vivo* metabolite profiling

Fecal and tissue homogenates were maintained at approximately −70°C for MET-ID analysis. The metabolite profiling in the biological samples from mice dosed with [14C]CA102N was performed by HPLC-tandem mass spectrometry coupled with a radio flow-through detector (RFD). The HPLC-MS/radio flow-through detector (RFD) consisted of a Shimadzu NexeraTM (Shimadzu, disseminated Japan) ultra-high pressure liquid chromatography system with two LC-30AD pumps, a SIL30AC autosampler, and a CTO-30A column oven; A Sciex TripleTOF 6600TM (Sciex, Framingham, MA) mass spectrometer; and a β-RAM Model 3 RFD. The mass spectrometer was controlled by Analyst TF 1.7TM (Sciex, Framingham, MA), and the RFD was controlled by Laura Lite 5TM (LabLogic Systems, Brandon, FL) software. The HPLC eluent was split between the RFD and mass spectrometer with a ratio of 3 to 1.

[14C]CA102N and its metabolites were separated using reverse-phase and hydrophilic interaction chromatography. Two HPLC methods were used to separate the metabolites and the parent molecule CA102N, using RFD and mass spectrometer simultaneously.

Retention times of the metabolites were determined from the radio-chromatograms. Mass spectra (MS and MS/MS) of metabolite standards (H-Nim, H-Nim Saccharide, H-Nim Disaccharide) were acquired and major fragment patterns were proposed. The identification of these metabolites was confirmed by matching mass spectra (MS and MS/MS) and retention times with authentic reference standards. For other unknown metabolites, molecular ions were searched on LC-MS chromatograms at the same retention times as those found on an HPLC radio-chromatogram. The product/ion spectra were acquired for the corresponding molecular ions. Putative metabolite structures were proposed based on the analysis of their mass fragment patterns.

### 2.7 PK of the parent molecule H-Nim in mice

Female BALB/c athymic (nu+/nu+) mice (n = 5) were used in the study. Mice were treated with a single bolus injection of H-Nim via the tail vein at 4.5 mg/kg. The blood samples were collected at 5, 10, 30, 60, and 120 min after treatment. Mice were anesthetized and decapitated to obtain the blood samples. Serum supernatant was collected and stored at −80 until analysis.

PK samples were analyzed by LCMS analysis. The retention time of H-Nim was approximately 25.4 min on the extracted ion chromatogram (XIC) and the protonated molecule of H-Nim was at m/z 279. The product ion spectrum of H-Nim displayed fragment ions at m/z 200, 171, 154 and 123.

### 2.8 Data analysis

Statistical analyses were limited to generating mean, standard deviation, and coefficient of variation calculations, as appropriate. Dose tables and data tables were compiled with the mean and standard deviation values calculated using the Debra^®^ software program (v. 6.06.63, Lablogic Ltd.) and/or Microsoft^®^ Office Excel 2010 (Microsoft Corporation).

The following calculations were used to determine the µg equivalents/mL, or/g and % percent of dose in the tissue:

µg equivalentsmL=µCimLSpecific Activity DosedµCimL 

µg equivalentsg=DPM of the sample×1MassTissuegSpecific Activity of the radiolabeled moleculeDPMg 

Percent of dose in tissue=µg eq in tissue×100Specific Activity DosedμCiμg

Note that: µg eq in the tissue=µg eq/g tissue×1g tissue=µg eq.

$$\text{Mass Balance Calculations}=\frac{Total\;\mu_{Ci}in\;sample}{Total\;\mu_{Ci}\;Dose}\times 100$$

#### 2.8.1 Plasma pharmacokinetic analysis

Pharmacokinetic parameters were determined using individual (Group 2) plasma total radioactivity concentration of [14C]CA102N and its metabolites vs. time profiles. Nominal times were used for pharmacokinetic calculations. Phoenix^®^ WinNonlin^®^, (version 6.3, Pharsight Corporation, Mountain View, CA, United States) noncompartmental pharmacokinetic analysis was used to determine PK parameters [Maximal concentration (C_max_), Time of maximal concentration (T_max_), Terminal half-life (t_1/2_), area under the curve to infinity observed (AUCinf obs [µg equiv·h/g]), and area under the curve all observed points (AUC_all_ [µg equiv·h/g])]. The acceptance criteria for the terminal half-life (t_1/2_) estimation used regression of at least three time points. Individual and/or mean plasma concentrations below the lower limit of quantitation (reported as BQL) were treated as zero for the calculation of mean concentration and standard deviation. Plasma concentrations are expressed as µg equivalents (equiv)/g (assume 1 mL = 1 g).

#### 2.8.2 QWBA calculations

Autoradioluminographic image data were calibrated using response curves that were generated using a weighted, 1st-degree, polynomial, linear equation (1/MDC/mm^2^). A numerical estimate of the goodness of fit was given by the relative error, where the absolute value for the relative error of each calibration standard must have been ≤0.250 to be accepted.

The LLOQ and ULOQ were set using calibration standard concentration values of 0.0003 and 20.973 μCi/g, respectively, which were widely proven values (Kolbe and Dietzel, 2000) that were verified during the QWBA system validation (Solon and Lee, 2002).

## 3 Results

### 3.1 Mass balance studies

**FIGURE 1:**
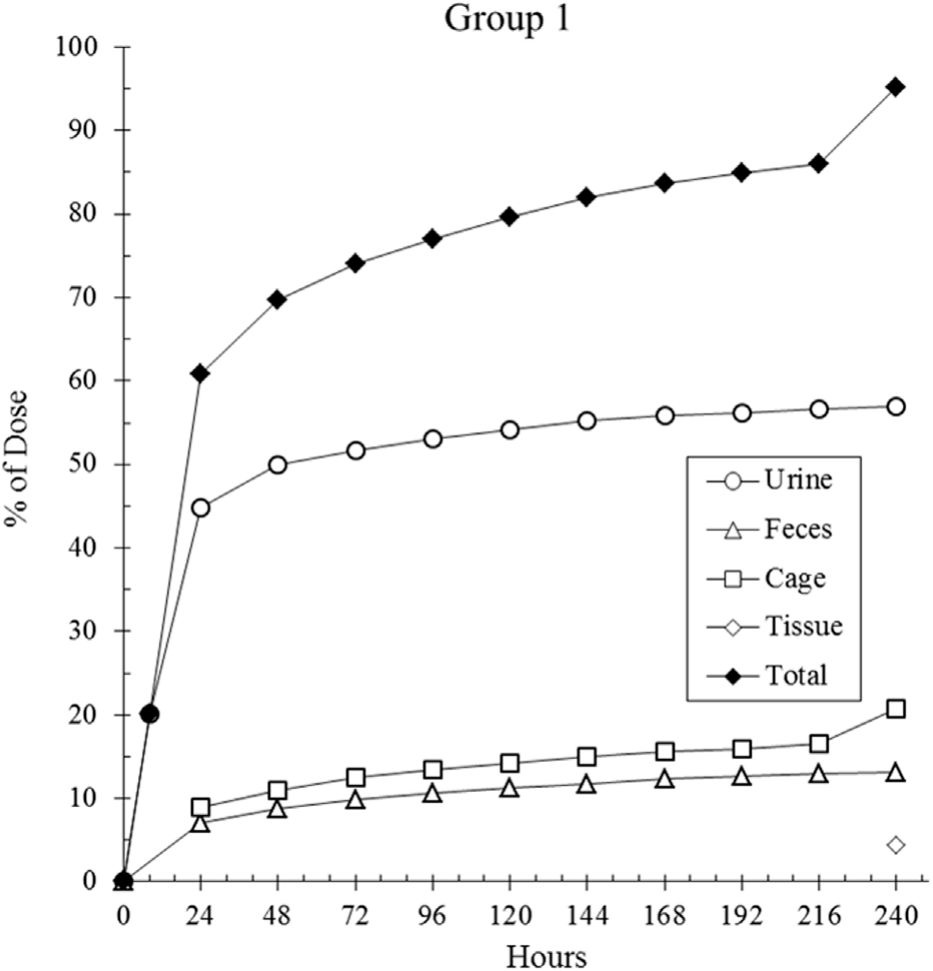
The cumulative recovery of total radioactivity over time. Total radioactivity was measured following a single intravenous (IV) dose of [14C] CA102N at 200 mg/kg in Group 1 tumor-bearing mice (n = 9). The data indicated that 94.7% of the administered radioactivity was recovered by 240 h post-dose, demonstrating significant retention and clearance of the compound/metabolites over time.

The primary route of elimination of CA102N after a single IV dose of [14C]CA102N in female BALB/c athymic (nu+/nu+) xenograft mice (Group 1) was in the urine, which accounted for a mean of 56.7% of the pooled administered dose. About 13.2% of the pooled administered dose was recovered in the feces. An average of 16.4%, 3.0%, and 1.2% of the pooled administered dose was recovered in the cage rinse, wash, and wipe, 120 h post-dose respectively. An average of 4.4% of the pooled administered dose was recovered in the tissues (i.e. liver and tumor, 240 h post-dose) that were collected from the mass balance animals. The average total recovery of radioactivity in mice was 94.9% of the pooled administered dose (Figure 1).

The highest tissue concentrations (>400 μg equiv/g) in were found in the following tissues: blood (1,458.127 μg equiv/g at 0.5 h), lung (884.995 μg equiv/g at 0.5 h), urinary bladder content (8984.365 μg equiv/g at 0.5 h), tumor (875.26 μg equiv/g at 8 h), liver (642.95 μg equiv/g at 48 h), adrenal Gland (429.979 μg equiv/g at 72 h), thyroid (469.62 μg equiv/g at 2 h), skin (non-pigmented) (407.673 μg equiv/g at 0.5 h), uterus (432.78 μg equiv/g at 0.5 h), and lymph node (456.819 μg equiv/g at 72 h) (Table 1).

**TABLE 1:** Distribution profile of total radioactivity (μg equivalents per gram tissue) determined by QWBA in tumors and major mouse tissues after i.v. administration of 200 mg/kg [14C] CA102N to the tumor-bearing mice (n = 1 per time point).

	Tissue (µg equiv/g)							
Time (h)	Lung	total tumor	Bone Marrow	Lymph Node	Spleen	kidney	Liver	Heart (myocardium)
0.5	885.00	608.05	260.58	162.05	185.07	961.30	199.26	246.03
2	701.41	540.81	244.14	386.80	162.19	431.35	176.74	174.40
8	316.66	875.26	196.36	440.87	186.54	229.05	299.67	88.89
24	92.67	790.25	313.36	557.33	263.57	82.75	441.21	42.59
48	2.65	534.73	393.32	216.96	322.28	50.10	642.95	16.04
72	2.88	336.54	322.01	522.57	331.05	40.04	531.83	10.75
120	0.74	278.65	237.90	414.03	109.73	17.70	283.96	7.15
168	0.73	188.86	204.72	423.70	97.28	16.95	274.63	3.25
240	0.48	134.33	185.87	398.62	120.64	13.67	202.64	2.92
336	0.47	31.22	145.98	243.38	88.72	7.15	193.85	1.61

LLOQ, 0.244 µg equiv/g tissue.

The tissues having the lowest relative concentrations (<60 μg equiv/g) were: white adipose, brain, spinal cord, bone tissue, eye lens, mammary gland region, and skeletal muscle.

### 3.2 Quantitative Whole-Body Autoradiography (QWBA) studies

**FIGURE 2:**
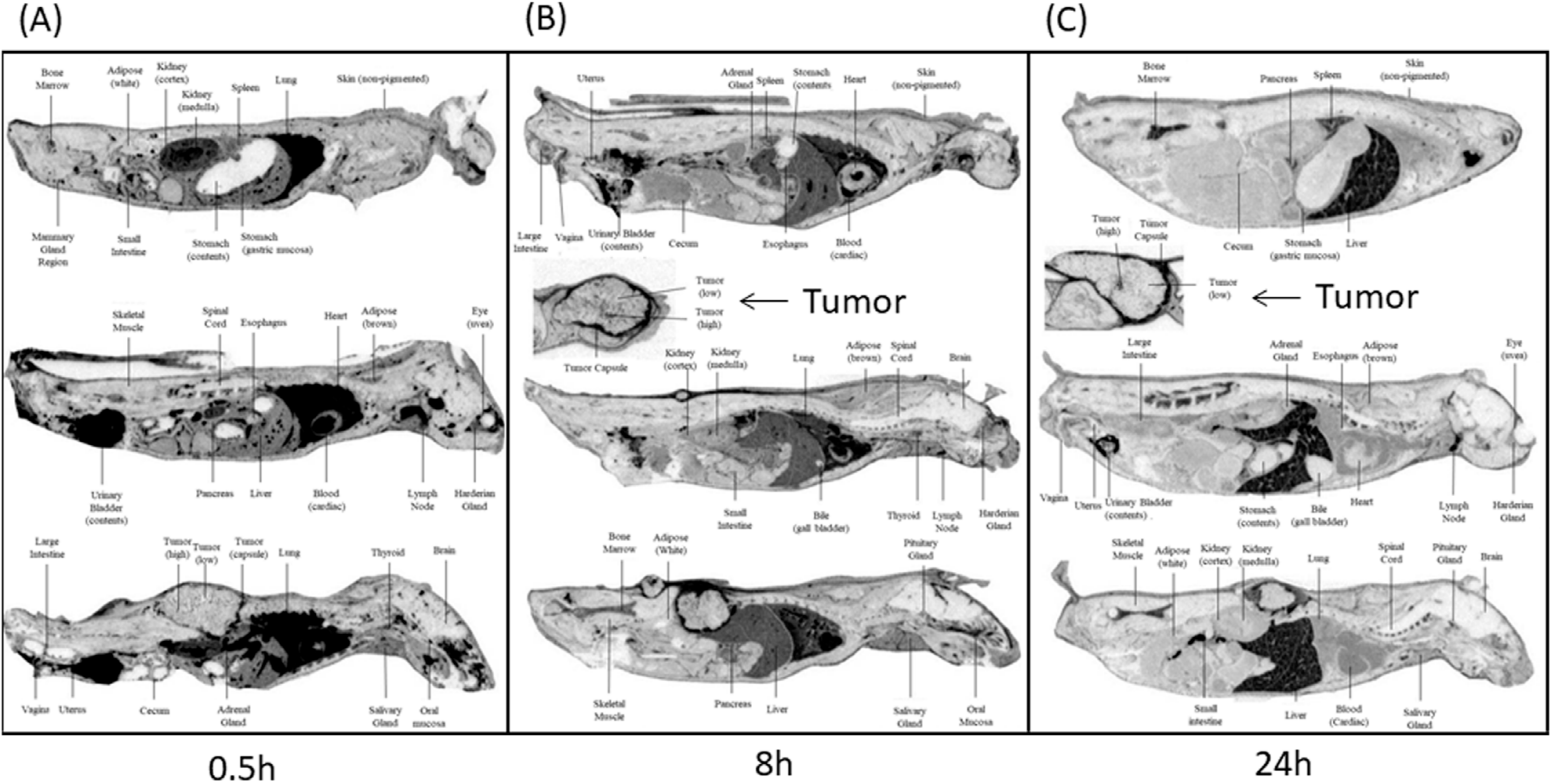
Whole-body autoradiograms (QWBA) of selected cross-sections of tumor-bearing mice. Selected cross-section tissues were taken at different time points **(A)** 0.5 h, **(B)** 8 h, and **(C)** 24 h post-administration of [14C] CA102N. Darker shades in the autoradiograms represent areas with higher concentrations of radioactivity, indicating increased drug distribution. Tumor areas are marked, and comparisons of radioactivity distribution between tumor tissues and other selected organs (e.g., liver, kidneys, lungs) are shown at each time point. The images demonstrate the temporal distribution of CA102N and metabolites, highlighting initial uptake and prolonged retention in tumor tissues. h refers to hours post-dose.

The mean tissue distribution in female athymic BALBc Nu+/Nu + HT-29 xenografted mice was examined following the administration of a single intravenous (IV) dose of ^[14C]^CA102N mixture (200 mg/kg). The concentration-time profiles of major tissues are summarized in Table 1 and and shown in the related autoradiograms (Figure 2). The [14C]CA102N and metabolites-derived radioactivity was well distributed in tissues. Most tissues exhibited lower concentrations than in blood at early time points (Supplementary Table S1); however, at time points 24 h and after, concentrations in blood continued to drop steadily, whereas concentrations in most tissues declined slowly and remained higher than in blood and some tissues (lymph node, bone marrow, spleen, liver, and tumor) for the remainder of the study. the C_max_ of [14C]CA102N and metabolites-derived radioactivity was observed in most tissues at 0.5 or 2 h post-dose, with tissue concentrations generally ≥80 μg equiv/g (Supplementary Figure S3).

### 3.3 Mice pharmacokinetics (PK)

**FIGURE 3:**
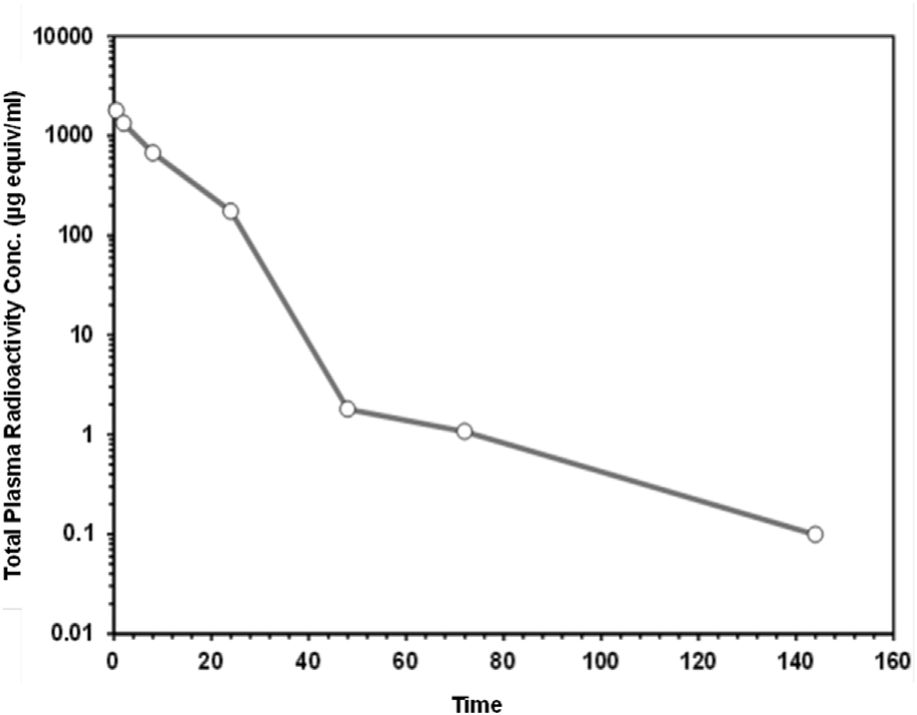
The mean plasma total radioactivity concentration data ([14C] CA102N and related molecules) versus time profiles following a single intravenous dose of [14C] CA102N at 200 mg/kg. Total plasma radioactivity in female BALB/c Athymic (nu+/nu+) mice (n = 5) was measured following a single intravenous (IV) dose of 200 mg/kg [14C]CA102N. Data points represent the mean ± standard deviation (SD), over time plotted on a logarithmic scale to show detailed kinetics throughout the study.

The plasma concentration-time data for, [14C]CA102N and its metabolites, was characterized in the plasma of female BALB/c athymic (nu+/nu+) xenograft mice following a single IV dose of [14C]CA102N equivalent to 200 mg/kg. The mean total radioactivity concentration-time data of CA102N and related molecules are shown in Figure 3. Pharmacokinetic parameters were determined using total radioactivity concentrations in plasma vs. time profiles.

The mean C_max_ of plasma total radioactivity after a single IV dose of [14C]CA102N and its metabolites was 1,798.586 μg equiv/ml. The T_max_ at C_max_ was observed at 0.5 h post-dose. Mean plasma radioactivity concentrations decreased over time with a t_1/2_ of 22 h. The mean plasma total radioactivity AUClast was 18,332.4 μg equiv/ml*h (Table 2). The results showed a rapid decline of total radioactivity concentrations after C_max_, 0.5 h post-dose. Low concentrations of CA102N and metabolites were quantifiable in plasma up to 144 h.

**TABLE 2:** Pharmacokinetic parameters total radioactivity concentration-time data ([14C]CA102N and metabolites) following a single intravenous administration of a nominal 200 mg/kg of [14C]CA102N in group 2 tumor-bearing female BALB/c athymic (nu+/nu+) mice.

Parameter	Unit	Value
Lambda_z	1/h	0.03
t_1/2_	h	22.4
T_max_	h	0.5
C_max_	μg equiv/ml	1798.5
C0	μg equiv/ml	1985.6
Clast_obs/C_max_		5.5E-05
AUC 0-t	μg equiv/ml*h	18329.2
AUC 0-inf_obs	μg equiv/ml*h	18332.4
AUC 0-t/0-inf_obs		0.99
AUMC 0-inf_obs	μg equiv/ml*h^2	161062.0
MRT 0-inf_obs	h	8.7
Vz_obs	(mg)/(μg equiv/ml)	0.3
Cl_obs	(mg)/(μg equiv/ml)/h	0.01
Vss_obs	(mg)/(μg/mL)	0.09

Mean dose administered: 197.27, SD, 13.18. N = 5 for all groups, except where noted; BQL, below the lower limit of quantitation; ND, not determined; SD, Standard Deviation. Abbreviations: AUC, the area under the concentration-time curve; C_max_, maximum concentration; t½, elimination half-life; T_max_, time to reach C_max_.

The PK analysis of H-Nim (parent molecule) was not possible as H-Nim was only observed at the first sampled time points of up to 5 min, (0.142 μg/mL). H-Nim was not detectable at any time point greater than 5 min (detection limit, 0.1 μg/mL). These results confirm the relatively short half-life and fast elimination of H-Nim.

### 3.4 Metabolite profiling of plasma, urine, fecal, liver, and Tumor extracts from female xenograft mice

#### 3.4.1 HPLC/MS analysis of metabolite standards

The identity of CA102N metabolites was confirmed by comparing the obtained mass spectra and retention times with the mass spectra and retention times of the reference standards, H-Nim, H-Nim Saccharide, and H-Nim Disaccharide.

### 3.4.2 Metabolite profiling

**FIGURE 4:**
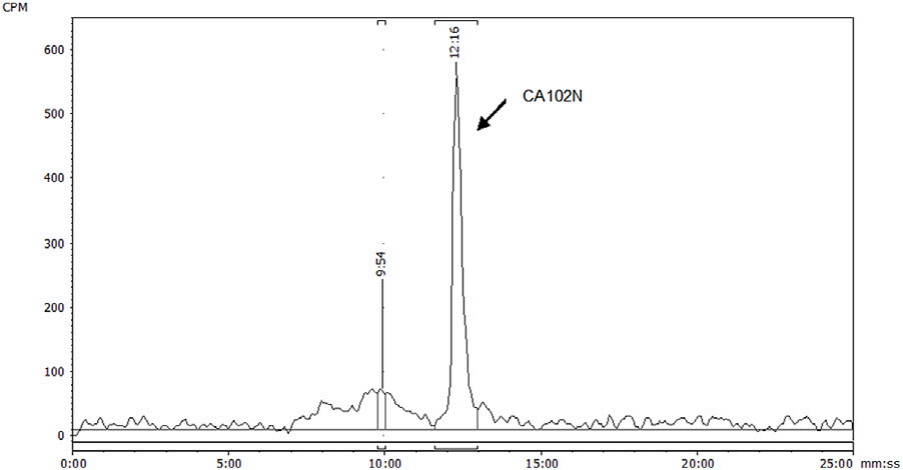
HPLC radio-chromatogram of plasma extracts from female BALB/c Athymic (nu+/nu+) xenograft mice following a single intravenous dose of [14C] CA102N at 200 mg/kg. HPLC radio-chromatogram displays the analysis of plasma extracts from female BALB/c Athymic (nu+/nu+) xenograft mice, pooled from 0 to 24 h following a single intravenous dose of [14C] CA102N at 200 mg/kg. The chromatogram demonstrates the presence of radiolabeled components, with the predominant peak corresponding to the parent compound, CA102N, indicating that the parent drug remains the predominant component in plasma during this period.

The recoveries of radioactivity from the LC column during a gradient run, determined from an injection of processed samples were all >90%. The radioactivity recoveries from mouse urine centrifugation were 99.06%. For the metabolite profiling, the identity of CA102N metabolites was confirmed by comparing the obtained mass spectra and retention times with the mass spectra and retention times of the reference standards, H-Nim, H-Nim saccharide, and H-Nim disaccharide. The parent molecule CA102N was the major product observed in the pooled plasma samples up to 24 h post-dose (Figure 4). CA102N is cleaved at the specific target site and two major metabolites, the H-Nim disaccharide conjugate and H-Nim tetrasaccharide, M1037 conjugate are released. No free Nim or H-Nim are determined in all the biological samples evaluated. This implies that the primary metabolic reaction likely involves glycosidic bond cleavage between sugar moieties or other linked components of the molecule.

**FIGURE 5:**
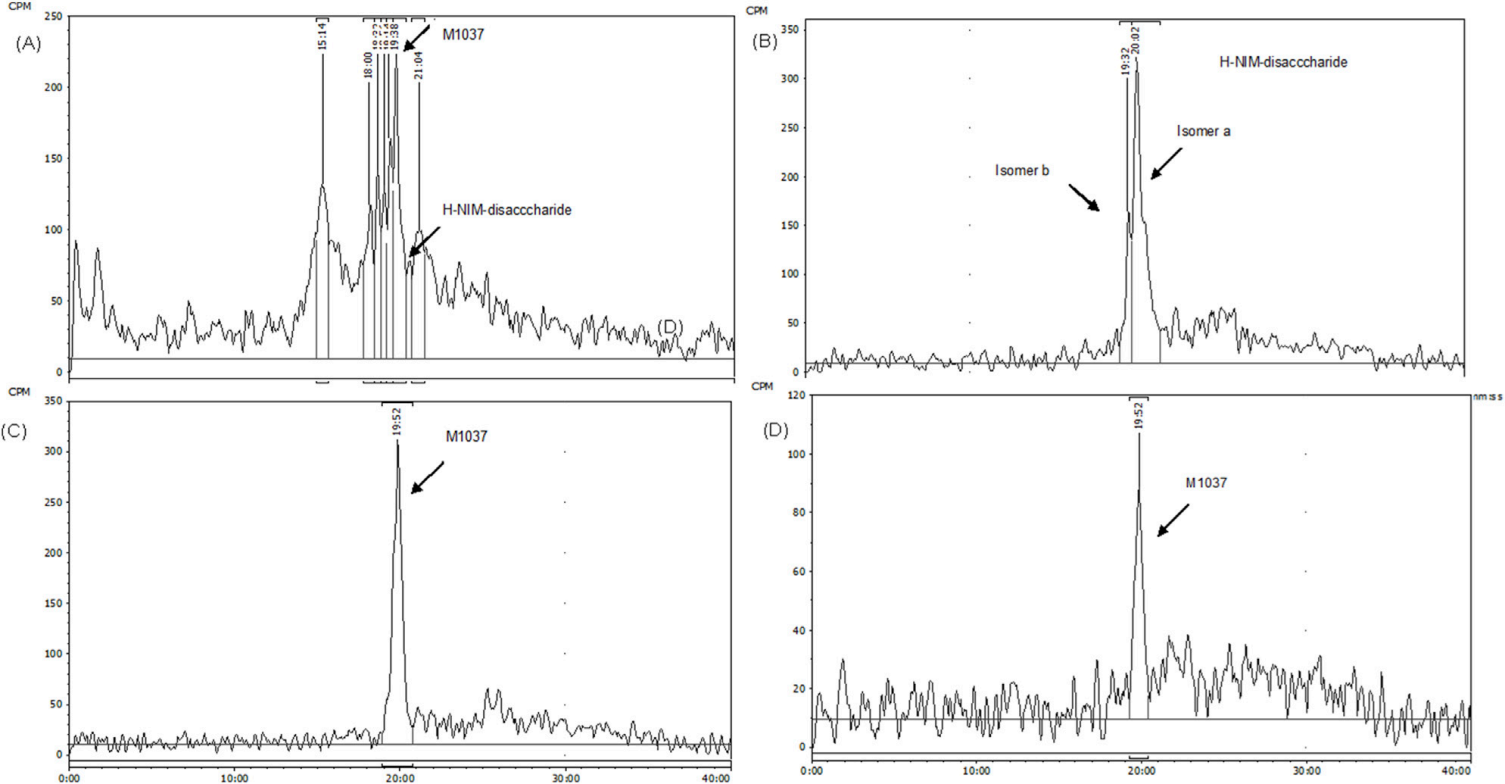
The HPLC Radio-chromatograms in different biological matrices of Female Xenograft Mouse. The radio-chromatograms display the presence of CA102N metabolites, specifically H-Nim-disaccharide (m/z 640) and M1037, in pooled mouse urine **(A)** and fecal extracts **(B)** collected from 0 to 120 h post-dose. Additionally, the metabolites were identified in the liver **(C)** and tumor tissues **(D)** at 240 h post-dose, confirming their tissue-specific distribution and retention.

**FIGURE 6:**
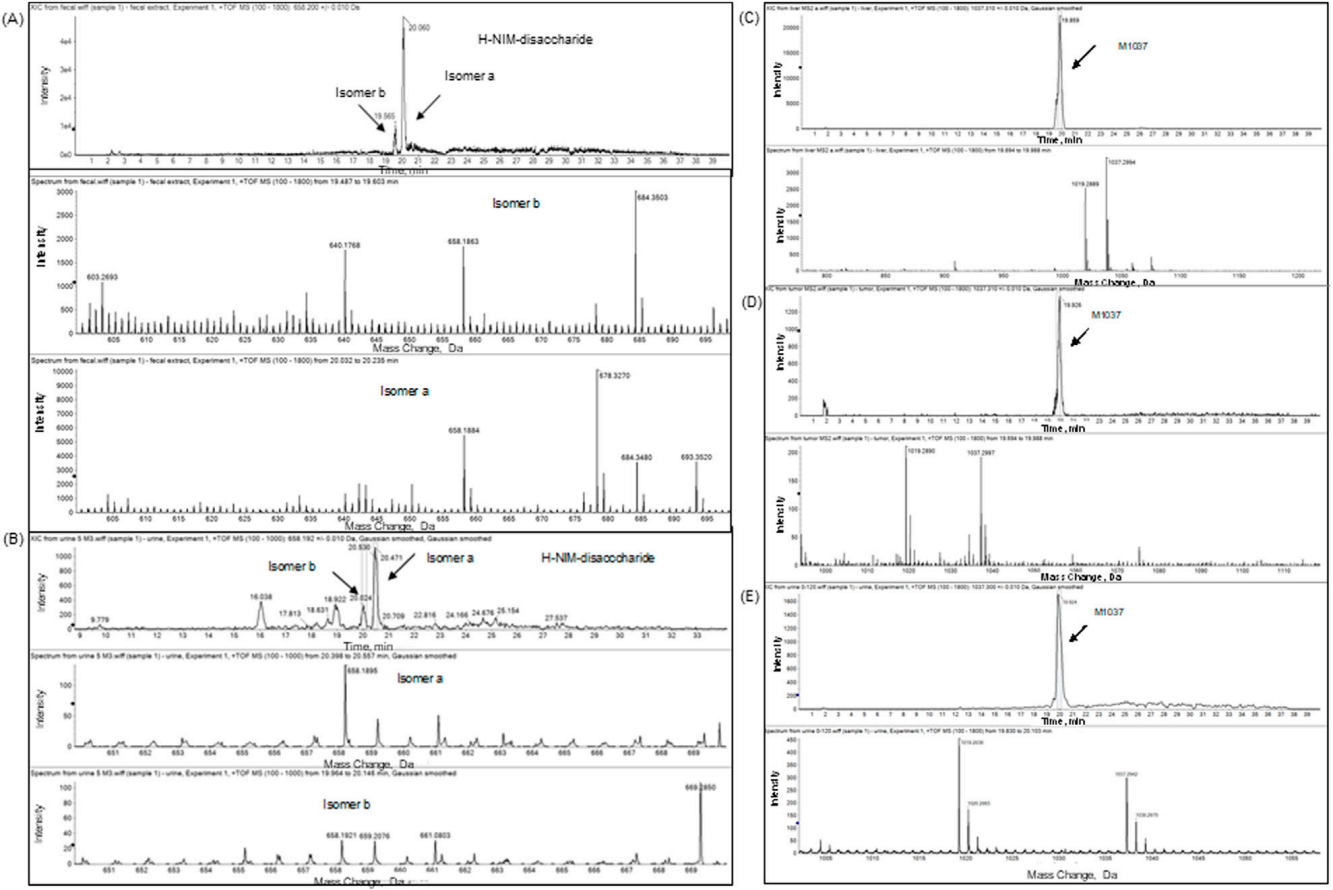
Extracted-ion chromatogram (XIC) and full scan mass spectrum of CA102N metabolites H-Nim-disaccharide and M1037 in different biological matrices. The top panels present the extracted-ion chromatograms (XIC), and the bottom panels display the full scan mass spectra for CA102N metabolites analyzed in various biological matrices. **(A, B)**: H-Nim-disaccharide identified in pooled mouse fecal extracts and urine samples collected from 0 to 120 h post-dose. The XIC shows a clear peak for H-Nim-disaccharide with a protonated molecule at m/z 640, and the corresponding full scan mass spectrum reveals characteristic fragment ions supporting the metabolite’s identity. **(C–E)**: M1037, a tetrasaccharide metabolite, was detected in liver and tumor tissues at 240 h post-dose and in urine samples pooled from 0 to 120 h post-dose.

H-Nim disaccharide was detected (isomer a and isomer b) in mouse fecal extracts and urine with a protonated molecule at m/z 658 (Figures 5A, B; Figures 6A, B; Supplementary Figures S4 A, B). The accurate mass of H-Nim disaccharide isomers were 658.1884 and 658.1863 Da, which matched the theoretical mass of 658.1913 Da for a formula C_27_H_36_N_3_O_14_^+^ (mass difference, −2.9, −7.6 ppm). The product ion spectra of H-Nim disaccharide standard isomers were similar and displayed prominent fragment ions at m/z 640, 437, 419, 358, 340, and 322. Both the retention time and product ion spectrum of H-Nim disaccharide matched the retention time and product ion spectrum of H-Nim disaccharide authentic standard.

The second metabolite, M1037, was identified in mouse liver and tumor extracts, and pooled urine samples with a protonated molecule at m/z 1,037 (Figures 5A, C, D; Figures 6C–E; Supplementary Figure S4C–E). The accurate mass of M1037 was 1,037.2994 Da, which matched the theoretical mass of 1,037.3027 Da for a formula C41H57N4O25+. The product-ion spectrum of M1037 displayed prominent fragment ions at m/z 1,019 (loss of water), 640, and 437. Based on the mass spectral data, M1037 was proposed as an H-Nim tetrasaccharide. The difference between the measured and theoretical masses is 0.0033 Da, which indicates a very close match, showing high confidence in the identification of this metabolite.

### 3.4.3 Biotransformation products of [14C]CA102N

**FIGURE 7:**
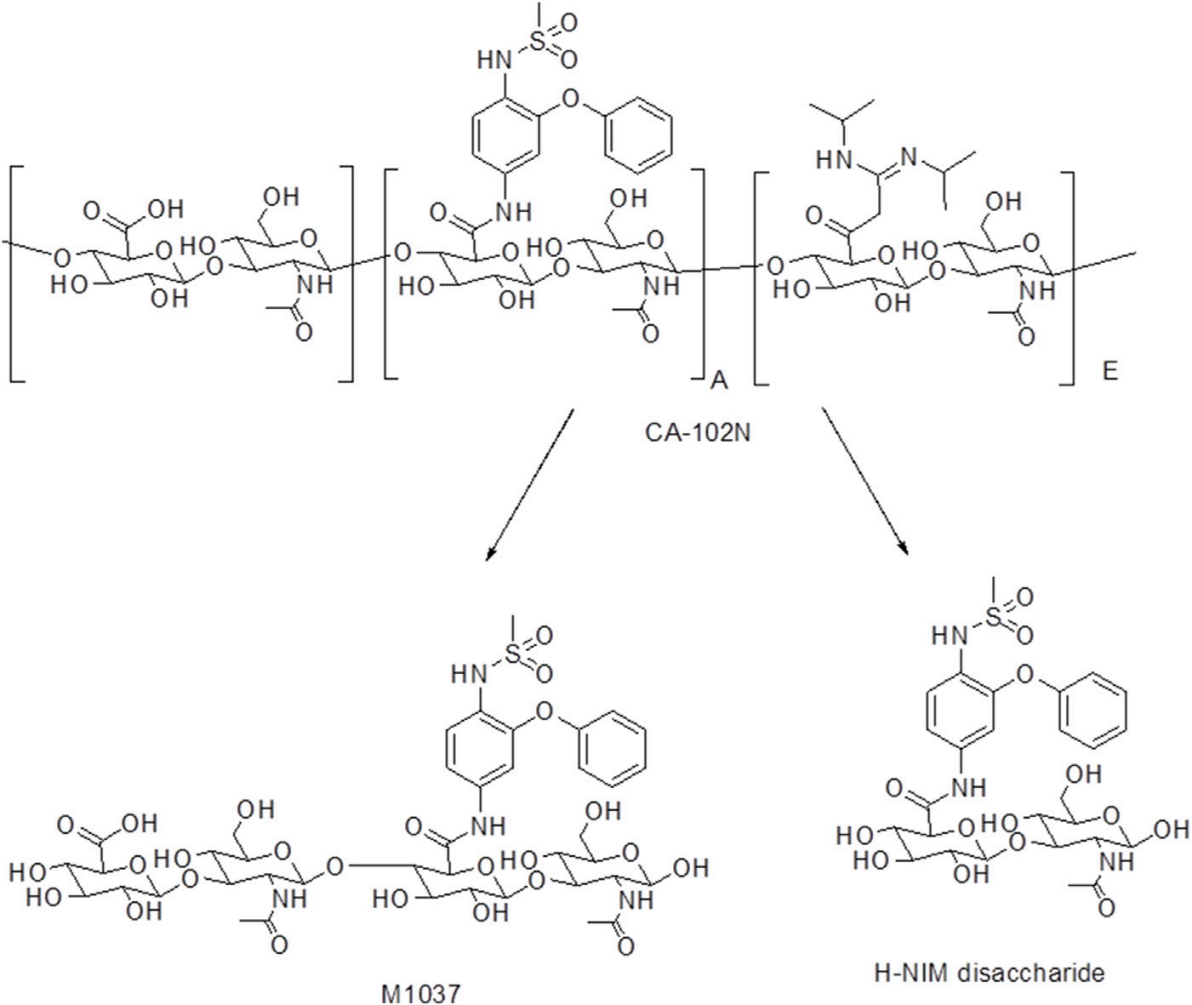
The biotransformation products of CA102N identified in female xenograft mice samples. CA102N metabolites (Nim-disaccharide and Nim-tetrasaccharide conjugates) are formed potentially by hydrolysis of glycosidic linkages within CA102N, leading to stepwise degradation into smaller saccharide units like disaccharides and tetrasaccharides.

The biotransformation products of [14C]CA102N in female xenograft mice included hydrolysis products H-Nim disaccharide and M1037, H-Nim tetrasaccharide (Figure 7).

## 4 Discussion

CA102N is an innovative anticancer agent of a new class of therapeutics based on the HADC platform, designed to selectively target tumors via the CD44 receptor overexpressed in many cancers. This novel platform exploits the interaction between HA and the CD44 receptor to optimize tumor-specific drug delivery (Mattheolabakis et al., 2015; Saravanakumar et al., 2014). For chemotherapeutics, receptor-mediated drug delivery is believed to reduce systemic toxicity and improve drug efficacy at tumor sites (Mohanty et al., 2011; Large et al., 2019). CA102N is an HA-H-Nim conjugate developed to deliver H-Nim to target cells to improve the overall safety, efficacy, and PK characteristics of the drug. Nimesulide is approved (in some countries) as a second-line treatment for acute pain, symptomatic treatment of painful osteoarthritis, and primary dysmenorrhea (Rainsford, 2006). Nimesulide at the recommended daily dose of 200 mg is associated with an increased risk for hepatotoxicity (HPRA, 2007; Page et al., 2008; Traversa et al., 2003). Besides, Nimesulide and H-Nim (a derivative of Nimesulide) often face challenges related to their poor solubility and pharmacokinetic (PK) profiles (Macpherson et al., 2013). In PK studies H-Nim, disappeared rapidly, showing a potential half-life of <5 min, limiting its druggability as a drug candidate. These limitations may require the development of alternative strategies to enhance solubility, stability, and PK profiles. Hyaluronic acid (HA) is a naturally occurring polysaccharide with excellent water-binding capacity and biocompatibility, making it an ideal carrier for drug conjugation. By conjugating HA with hydrophobic drugs such as H-Nim, the overall solubility of the drug can be significantly improved. Additionally, HA as a carrier helps to prolong the circulation time of the drug, since the conjugate is less likely to be cleaved in the bloodstream. This also allows for controlled release and delivery to target tissues, such as tumors, where enzymes like hyaluronidases break down the HA and release the potential drug, metabolites. Therefore, the conjugation of H-Nim with HA in CA102N not only provides enhanced solubility, which is crucial for its parenteral administration but also improves its pharmacokinetics and tumor-targeting capabilities.

Understanding the PK and DME characteristics of a molecule is key to optimizing therapeutic potential, reducing side effects, and ensuring safety and efficacy. The present study was designed to evaluate for the first time the systemic exposure, tissue distribution and the DME properties of CA102N (H-Nim), an HADC, using a human colon carcinoma (HT-29) xenograft mouse model. The study was conducted in part to validate the HADC drug carrier system, highlighting its potential to improve drug targeting, delivery efficiency, and PK profile. Xenograft models were used in this study for they are often employed in cancer research as valuable models for understanding drug behavior *in vivo* and for confirming tumor-targeting properties of drug candidates prior to clinical trials (Kelland LR, 2004; Ruggeri et al., 2014). Although the data obtained from xenograft models may differ from human studies, it can still offer valuable insights for subsequent human experiments. Preclinical data from such models can guide dose selection and predict potential tissue-specific accumulation. These assessments are critical for designing human studies in clinical trials. In addition, in this study radiolabeled [14C]CA102N was used, with the radiolabel attached to the aniline ring of the H-Nim moiety. This approach facilitated the tracking of both the parent drug and its metabolites across various tissues and organs, which enabled the determination of its biodistribution.

Pharmacokinetic analysis of total radioactivity following a single intravenous (I.V.) dose of 200 mg/kg [14C] CA102N included both CA102N and its circulating metabolites. The maximum concentration (C_max_) of 1798.58 µg equivalents/mL was reached within 0.5 h post-administration (T_max_). The plasma concentration-time profile demonstrated a rapid distribution phase followed by a slower elimination phase, likely due to the gradual release of CA102N from cellular compartments. At 72 h post-dose, the mean plasma radioactivity was 1.073 µg equivalents/mL. CA102N showed a low systemic plasma clearance (CL) of 0.012 L/h/kg and a volume of distribution at a steady state (Vss) of 0.1 L/kg, which is smaller than the total body water volume in mice (0.7 L/kg). This suggests that CA102N may preferentially penetrate tissues with high CD44 expression.

The observed half-life (t_1/2_) of 22 h, a contrast to the much shorter half-life of the parent molecule H-Nim (t_1/2_ < 5 min), suggests that conjugation significantly enhanced the pharmacokinetic profile of H-Nim. Moreover, conjugation most likely improved the molecule’s stability and solubility, which could further potentiate the therapeutic impact of CA102N. By remaining bioavailable in the system for a much longer period, CA102N may also allow for sustained pharmacological action, potentially leading to enhanced therapeutic efficacy.

The QWBA imaging data provided valuable insights into the tissue distribution and retention of CA102N and active metabolites in tumor-bearing mice following intravenous administration. Measurable levels of CA102N and its associated molecules were detected in various tissues at all observational time points, highlighting the compound’s systemic exposure and targeted delivery capabilities. The highest total radioactivity concentrations were found in cardiac blood, lung, liver, kidney medulla, and the tumor capsule at T_max_, indicating key tissues where the drug and/or its metabolites accumulated.

The immediate and significant tumor accumulation (12.41% of the total dose at 0.5 h post-dose and a C_max_ of 17.86% after 8 h) suggests that CA102N is rapidly delivered to tumor sites. This tumor targeting most possibly occurs through two primary mechanisms: CD44 Receptor-Mediated Targeting and the Enhanced Permeation and Retention (EPR) Effect. High CD44 expression in tumors enables selective binding and internalization of CA102N, making CD44-mediated targeting a critical factor for drug targeting and tumor selectivity. Additionally, the EPR effect likely played a crucial role in the increased tumor uptake and prolonged retention of CA102N (Maeda et al., 2016). The EPR effect allows passive accumulation of CA102N in tumors, where the drug remains for extended periods, as evidenced by the sustained release of CA102N and its metabolites from tumor sites, with a half-life of 74.2 h. This dual-targeting mechanism further enhances CA102N’s potential as a therapeutic agent.

The total recovered radioactivity of the administered dose was 94.9%, with 56.7% excreted in urine and 13.2% recovered in feces. The cage rinses, wash, and wipe accounted for a combined total of 20% of the dose. The primary source of cage rinse radioactivity (16.4%) was likely urine, suggesting that approximately 73% of the total [14C] CA102N-related material was excreted via urine (Table 3). Around 60% of the total dose was eliminated within the first 24 h, with the remaining amount likely being slowly released from tissues over time. Detectable levels of radioactivity were still present in certain tissues at the time of sacrifice, 10 days post-dose. The average residual radioactivity in liver and tumor tissues was 4.4% of the total administered dose, indicating prolonged retention in these tissues. The urinary excretion pattern aligns with the observed plasma half-life (CA102N and metabolites) of 22 h and its low clearance rate, supporting the hypothesis that CA102N-related molecules are released gradually from tissues over an extended period.

**TABLE 3:** Excretion of CA102N, M1, and M1037 and total radioactivity following a single 200 mg/kg intravenous injection of [14C] CA102N in group 1 mice.

Percent of dose Excretion (measured radioactivity)	Urine + (Cage Rinse)	Feces	Liver + Tumor	Total
	(56.7 ± 4.8)+ (16.4 ± 7.6)	13.20 ± 8	4.40 ± 0.3	94.90 ± 13.8
Excretion by component				
M1	ND	13.20%	ND	13.2%
M1+M1037	77%			77%
M1037		ND	4.40%	4.4%
CA102N	ND	ND	ND	ND

All data are means ± standard deviations (n = 9). ND, not detected.

While CA102N was the major component observed in mouse plasma, two additional metabolites were identified across various tissues, urine, and feces. The primary metabolite in fecal extracts was H-Nim disaccharide, whereas M1037 (H-Nim tetrasaccharide) was the dominant metabolite in liver and tumor extracts. Both metabolites were determined in pooled urine samples. The cleavage of HA or CA102N may be initiated by hyaluronidases, particularly the HYAL-2 enzyme, which breaks down HA into intermediate-sized fragments of about 10–20 kDa (Stern, 2003; Lepperdinger et al., 2004). These fragments are taken up by cells via endocytosis after binding to HA receptors such as CD44 (Harada and Takahashi, 2007). Once internalized, hyaluronidase HYAL-1 further degrades HA/CA102N into predominantly tetrasaccharides within the endosomal-lysosomal compartments. These tetrasaccharides could either be cleaved into disaccharides by lysosomal glucuronidases or released intact through exocytosis (Jourdian, 1996; Racine and Mummert, 2012; Winchester, 1996).

No Nim-related metabolites or degradation products were observed during the study, and there was no indication of toxicity linked to Nim or its derivatives. This suggests that the conjugation of H-Nim to hyaluronic acid (HA) in CA102N may have altered its metabolic profile, reducing the formation of potentially toxic Nim-related compounds. Therefore, the metabolic profile, improved pharmacokinetics, and targeted delivery of CA102N via HA-CD44 interaction, likely contributed to its favorable safety profile. These findings are consistent with the absence of hepatotoxicity observed in preclinical toxicology and clinical studies (Pant et al., 2023; Tchaparian et al., 2019).

One of the limitations of this study is the small number of HT-29 xenograft model mice (n = 1)/time point used in the biodistribution assessment. However, unlike *in vitro* and *ex vivo* assays of excised organs or tissues, QWBA measurements minimize intra-subject variability by enabling longitudinal tracking of tissue distribution across multiple time points. This approach enhances its statistical power and ensures efficient data acquisition with reasonably high reliability, despite the limited sample size.

Another limitation is that the metabolic profile of CA102N was unknown at the time of this study. Consequently, total radioactivity was used to estimate the overall clearance and pharmacokinetic (PK) parameters, which may not fully capture the exact metabolic pathways of CA102N. Further studies will be necessary for a full characterization of the biotransformation pathway of CA102N.

## 5 Conclusion

In summary, the current study provided a comprehensive understanding of the PK and DME characteristics of CA102N and related molecules in mice following a single intravenous dose of 200 mg/kg. The pharmacokinetic profile was thoroughly characterized, including its biodistribution across various tissues, with a particular emphasis on tumor accumulation. Urinary excretion was identified as the major elimination pathway of CA102N. CA102N remained the primary circulating component in plasma for up to 24 h while two metabolites, a disaccharide, and a tetrasaccharide, were identified in the excreta and tissue samples. The metabolites profile revealed interesting hydrolysis pathways with no detectable fragments of the parent molecule, H-Nim.

These studies were instrumental in identifying the PK DME properties of CA102N, assessing potential safety risks, and supporting dosing decisions. The findings contributed significantly to the overall development of CA102N, allowing its successful advancement as a drug candidate in clinical settings.

## Data Availability

The original contributions presented in the study are included in the article/Supplementary Material, further inquiries can be directed to the corresponding author.
