# Supplementary material for: Mass balance, metabolic disposition, and pharmacokinetics of a single IV dose of [14C]CA102N in HT-29 xenograft athymic nude mice
Source: Front Pharmacol. 2024 Dec 5;15:1440679. doi: 10.3389/fphar.2024.1440679 (PMC11655901; doi:10.3389/fphar.2024.1440679)
Supplement: Supplementary file 1 [file DataSheet1.pdf]

Supplementary Material

1 Supplementary Figures and Tables

1.1 Supplementary Figures.

Supplementary Figure 1. CA102N, Nim and H-Nim Chemical Structures

Supplementary Figure 2. Representative radio-chromatograms of Pre-dose and Post-dose of [14C]CA102N

Supplementary Figure 3. The concentrations-time course of [14C] CA102N and related molecules in various tissues

Supplementary Figure 4. LC-MS/MS identification of CA102N metabolites in urine (S4A and E), feces(S4B) and liver extracts (4C) or tumor(4D)

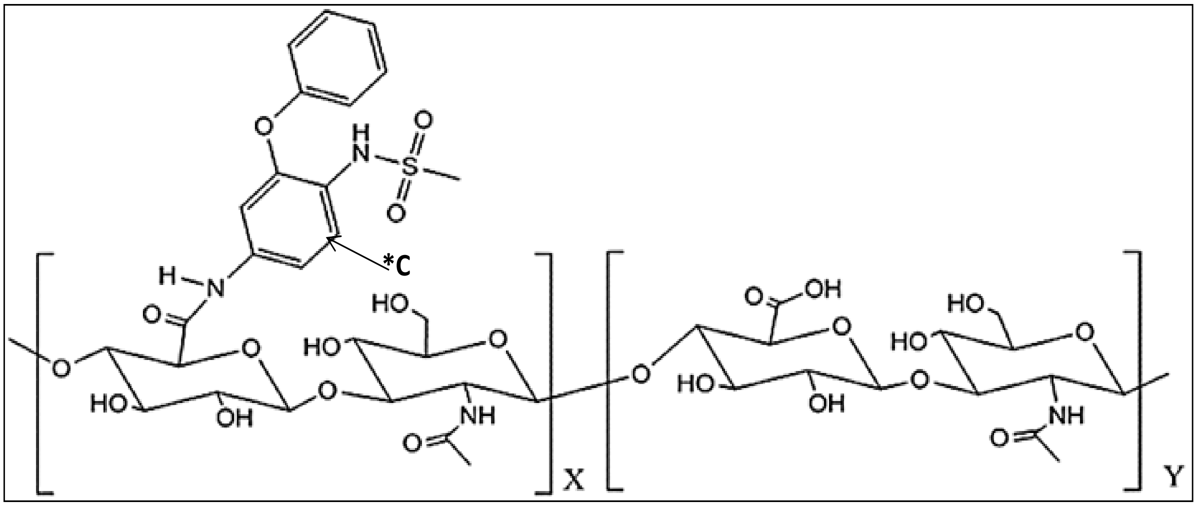

Nimesulide H-Nim

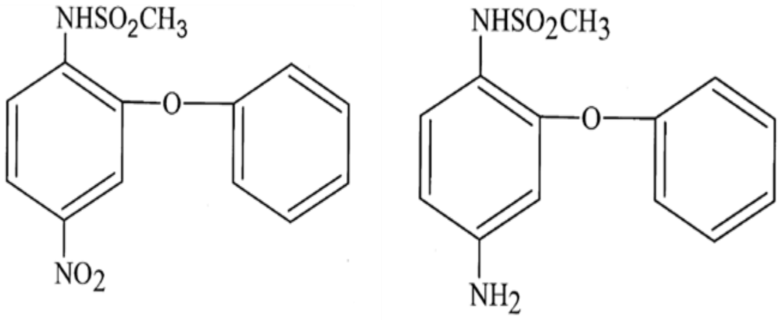

Supplementary Figure 1. CA102N, Nim and H-Nim Chemical Structures

X = number of hyaluronate carboxyl groups substituted with Nimesulide (Degree of Substitution) Y = number of unsubstituted hyaluronate disaccharides, Molecular Formula: (C27H33N3O13S)x - (C14H21NO11)y, Molecular Weight: about 150 kDa.

(A)

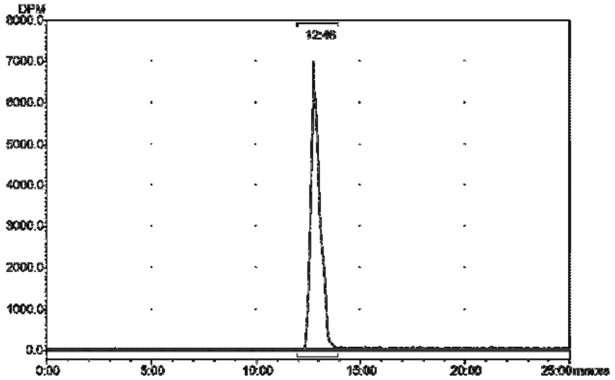

Regions: 14C Detector: ß-RAM

Name (C-14)	Start (mm:ss)	End (mm:ss)	Retention (mm:ss)	Height (DPM)	Area (DPM)	%ROI (%)
Region 1	11:58	13:54	12:46	6680.0	101868.0	100.00
1 Peak					101868.0	100.00

(B)

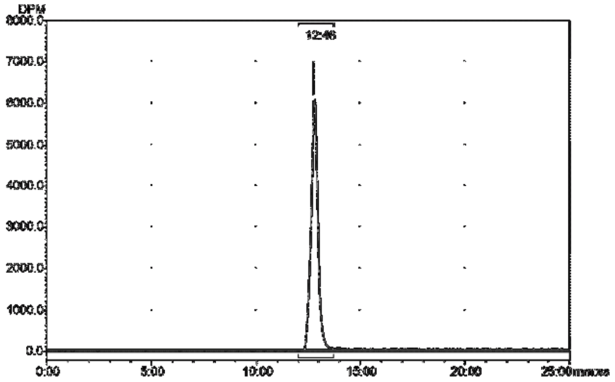

Regions: 14C Detector: ß-RAM

Name (C-14)	Start (mm:ss)	End (mm:ss)	Retention (mm:ss)	Height (DPM)	Area (DPM)	%ROI (%)
Region 1	12:02	13:42	12:46	6768.0	69450.0	100.00
1 Peak					69450.0	100.00

Supplementary Figure 2. Representative radio-chromatograms of Pre-dose and Post-dose of [14C]CA102N.

The radiopurity, homogeneity, and stability of the dosing formulation were assessed by analyzing a pre-and post-dose aliquot (23.5 µL) of the dosing formulation that was diluted to 1 mL with DPBS using the radio-HPLC method.

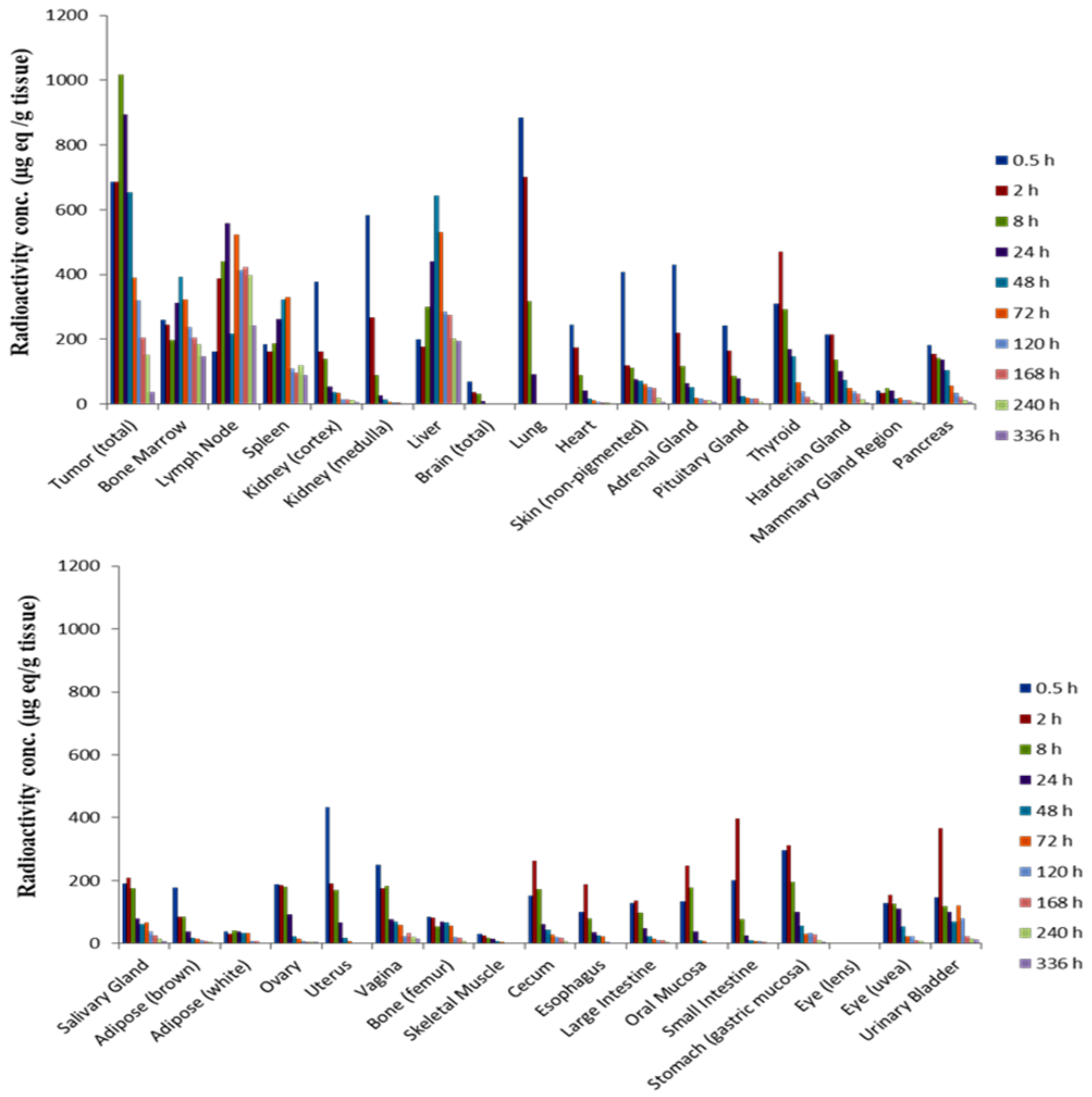

Supplementary Figure 3. The concentrations-time course of [14C] CA102N and related molecules in various tissues.

The concentration-time course of total radioactivity was measured in various tissues of female BALB/c Athymic (nu+/nu+) mice at 0.5, 2, 8, 24, 48.72, 120, 168, 240, and 336 hours following a single IV dose of 200 mg/kg (n = 1 per time point). The data indicate accumulation and retention of CA102N and its metabolites in specific tissues after administration, suggesting prolonged retention in target tissues, particularly tumors.

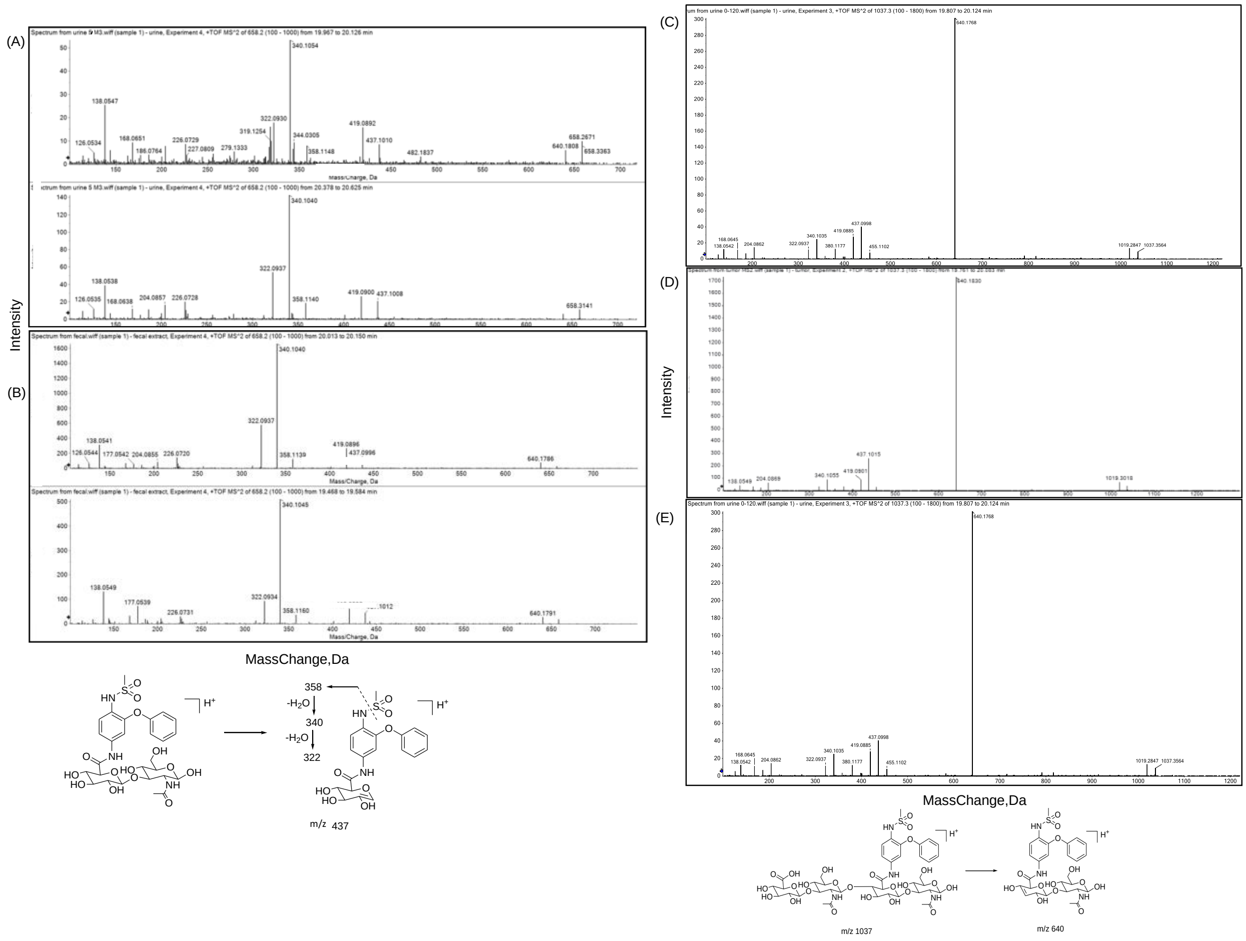

Supplementary Figure 4. LC-MS/MS identification of CA102N metabolites in urine (S4A and E), feces(S4B), and liver extracts (4C) or tumor(4D).

Two key metabolites, H-Nim tetrasaccharide (m/z 1037) and Nim-disaccharide (m/z 640) were detected in the pooled mouse urine, Nim-disaccharide (m/z 640) in fecal samples, while in liver and tumor extracts at 240 hours post-dose H-Nim tetrasaccharide (m/z 1037) was detected. The product ion spectrum further confirmed the identity of these metabolites, indicating the breakdown products of CA102N in vivo.

1.2 Supplementary Tables

Supplementary Table 1**.**Tissue-to-blood ratio measured in various tissues

Supplementary Table 1. Tissue-to-blood ratio measured in various tissues at different time points following a single 200 mg/kg IV bolus dose of CA102N

Tissue Type	Tissue	0.5 h	$t/b_{0.5}$	8 h	t/b_8	24 h	t/b_{24}	48h	t/b_{48}
Vascular/ Lymphatic	Blood (cardiac)	1458.13	1.00	552.12	1.00	119.50	1.00	1.50	1.00
	Bone Marrow	260.58	0.18	196.36	0.36	313.36	**2.62**	393.32	**3.29**
	Lymph Node	162.05	0.11	440.87	0.80	557.33	**4.66**	216.96	**1.82**
	Spleen	185.07	0.13	186.54	0.34	263.57	**2.21**	322.28	**2.70**
Excretory/ Metabolic	Kidney (cortex)	378.46	0.26	140.46	0.25	55.47	0.46	36.30	0.30
	Kidney (medulla)	582.84	0.40	88.59	0.16	27.29	0.23	13.79	0.12
	Liver	199.26	0.14	299.67	054	441.21	**3.69**	642.95	**5.38**
	Urinary Bladder	146.49	0.10	119.26	0.22	101.07	0.85	70.29	0.59
	Urinary Bladder (contents)	8984.37	**6.16**	1178.97	**2.14**	615.56	5.15	49.77	0.42
Endocrine	Adrenal Gland	429.98	0.29	116.47	0.21	64.39	0.54	50.89	0.43
	Pituitary Gland	242.97	0.17	86.17	0.16	78.75	0.66	25.20	0.21
	Thyroid	310.17	0.21	293.69	0.53	168.49	1.41	146.18	1.22
Secretory	Harderian Gland	215.77	0.15	137.01	0.25	101.75	0.85	74.48	0.62
	Mammary Gland Region	41.26	0.03	48.17	0.09	41.34	0.35	16.54	0.14
	Pancreas	182.22	0.12	142.54	0.26	137.93	**1.15**	103.53	0.87
	Salivary Gland	191.55	0.13	174.95	0.32	78.50	0.66	61.88	0.52
Fatty	Adipose (brown)	177.69	0.12	84.25	0.15	37.83	0.32	16.73	0.14

	Adipose (white)	37.69	0.03	40.06	0.07	38.71	0.32	33.67	0.28
Dermal	Skin (non-pigmented)	407.67	0.28	112.25	0.20	77.17	0.65	71.93	0.60
Reproductive	Ovary	188.87	0.13	180.77	0.33	91.37	0.76	23.01	0.19
	Uterus	432.78	0.30	169.95	0.31	66.05	0.55	17.70	0.15
	Vagina	249.67	0.17	182.17	0.33	76.75	0.64	69.95	0.59
Muscular	Heart (myocardium)	246.03	0.17	88.89	0.16	42.59	0.36	16.04	0.13
Respiratory Tract	Lung	885.00	0.61	316.66	0.57	92.67	0.78	2.65	0.02
Alimentary Canal	Cecum	153.07	0.10	173.46	0.31	62.67	0.52	44.12	0.37
	Esophagus	99.26	0.07	79.88	0.14	35.77	0.30	26.34	0.22
	Large Intestine	128.22	0.09	98.72	0.18	48.69	0.41	23.30	0.19
	Large Intestine (contents)	3.45	0.00	175.69	0.32	68.99	0.58	22.99	0.19
	Oral Mucosa	133.19	0.09	176.97	0.32	38.78	0.32	10.19	0.09
	Small Intestine	202.34	0.14	77.22	0.14	26.21	0.22	9.78	0.08
	Small Intestine (contents)	111.54	0.08	398.64	0.72	42.03	0.35	22.05	0.18
	Stomach (gastric mucosa)	296.90	0.20	195.44	0.35	99.67	0.83	56.13	0.47
	Stomach (contents)	2.97	0.00	6.96	0.01	23.27	0.19	8.02	0.07
Tumor	Total	78.38	0.05	141.46	0.26	102.94	0.86	118.62	0.99
	High	53.09	0.04	234.89	0.43	231.59	**1.94**	49.18	0.41
	Low	4.94	0.00	72.82	0.13	11.89	0.10	8.80	0.07

Capsule	550.02	0.38	567.56	1.03	546.77	**4.58**	476.75	**3.99**

Concentrations are in microgram per gram tissue (µg equiv/g tissue), tissue to blood ratio, t/b,. n=1/time point. Ratios.> 1 are indicated in bold. LLOQ = 0.244 µg equiv/g tissue.
